# Hearing Loss and Frailty Among Older Adults: The ARIC Neurocognitive Study

**DOI:** 10.1093/geroni/igaa057.2948

**Published:** 2020-12-16

**Authors:** Nicholas Reed, Emmanuel Garcia-Morales, Priya Palta, Frank Lin, Josef Coresh, Jennifer Schrack, Jennifer Deal

**Affiliations:** 1 Johns Hopkins University, Baltimore, Maryland, United States; 2 Johns Hopkins Bloomberg School of Public Health, Baltimore, Maryland, United States; 3 Columbia University Irving Medical Center, New York, New York, United States; 4 Johns Hopkins University School of Medicine, Baltimore, Maryland, United States

## Abstract

Hearing Loss (HL) is common among older adults and is associated with factors (e.g., walking speed and social isolation) that may mediate an association with frailty. In the Atherosclerosis Risk in Communities (ARIC) study, frailty was defined as a composite variable (unintentional weight loss, energy expenditure, walking speed, low energy, and grip strength) while HL was measured using pure-tone audiometry. Among, 3179 participants in 2015-2017, 251 (7.9%) were frail. In a model adjusted for demographic and clinical risk factors, mild HL (n=1263; Odds Ratio[OR]=1.42; 95%Confidence Interval[CI]=1.01-2.01) and moderate HL (n=854; OR=1.67; 95%CI=1.09-2.55) were associated with higher odds of frailty relative to those without HL (n=1063). Among participants who completed an ARIC visit 2-years later, the odds of developing frailty tended to be higher among those with mild (OR=1.46; 95%CI=0.91-2.33) and moderate HL (OR=1.43; 95%CI=0.77-2.67). Future research should focus on mechanisms underlying association and determine the impact of treatment of HL. Part of a symposium sponsored by Sensory Health Interest Group.

